# The Effects of Learning in Morphologically Evolving Robot Systems

**DOI:** 10.3389/frobt.2022.797393

**Published:** 2022-05-27

**Authors:** Jie Luo, Aart C. Stuurman, Jakub M. Tomczak, Jacintha Ellers, Agoston E. Eiben

**Affiliations:** Department of Computer Science, Vrije Universiteit Amsterdam, Amsterdam, Netherlands

**Keywords:** evolutionary robotics, embodied intelligence, modular robots, evolvable morphologies, lifetime learning, targeted locomotion

## Abstract

Simultaneously evolving morphologies (bodies) and controllers (brains) of robots can cause a mismatch between the inherited body and brain in the offspring. To mitigate this problem, the addition of an infant learning period has been proposed relatively long ago by the so-called Triangle of Life approach. However, an empirical assessment is still lacking to-date. In this paper, we investigate the effects of such a learning mechanism from different perspectives. Using extensive simulations we show that learning can greatly increase task performance and reduce the number of generations required to reach a certain fitness level compared to the purely evolutionary approach. Furthermore, we demonstrate that the evolved morphologies will be also different, even though learning only directly affects the controllers. This provides a quantitative demonstration that changes in the brain can induce changes in the body. Finally, we examine the *learning delta* defined as the performance difference between the inherited and the learned brain, and find that it is growing throughout the evolutionary process. This shows that evolution produces robots with an increasing plasticity, that is, consecutive generations become better learners and, consequently, they perform better at the given task. Moreover, our results demonstrate that the Triangle of Life is not only a concept of theoretical interest, but a system methodology with practical benefits.

## 1 Introduction

The interest of this paper is an evolutionary robot system where robot morphologies (bodies) and controllers (brains) are evolved simultaneously. Reproduction in such a system requires that a new body and a new brain are created and put together to form the robot offspring. In case of asexual reproduction, one parent is sufficient and a new body and a new brain are obtained by random variation (mutation). For sexual reproduction (crossover), the bodies and the brains of the parents need to be recombined and the offspring is made up by the resulting body, driven by the resulting brain.

Regardless the specific way of reproduction, in general it cannot be assumed that the newly produced body and the newly produced brain form a good match. Even though the parents might have well-matching bodies and brains, recombination and mutation are stochastic operators and can shuffle the parental genomes such that the resulting body and brain do not form a good combination. Thus, the joint evolution of morphologies and controllers inherently leads to a potential body-brain mismatch. This problem has been originally noted by (Eiben et al., 2013) and recently revisited in (Eiben and Hart, 2020). The proposed solution is the addition of learning. In terms of the robotic life cycle, this means an extension of the straightforward two stages, the Morphogenesis Stage before ‘birth’ (when the robotic phenotype is constructed according to the instructions in the genotype) and the Operational Stage after “birth” (when the robot is carrying out its task(s) and tries to reproduce). The third stage is the so-called Infancy Stage, following Morphogenesis, when the “newborn” robot is learning to optimize the control of the inherited body and realize its maximum potential. The name Infancy is not only metaphorical, it also indicates an important detail: during this stage the robot is not fertile, that is, not eligible for reproduction. Algorithmically, this means that the fitness of a new robot is only calculated after the Infancy period, thus the robot can be subject to the (fitness based) mate selection procedure only after the learning is finished.

This three-stage system methodology, dubbed The Triangle of Life, has been described in (Eiben et al., 2013), but to-date there are hardly any studies into the workings of such systems. One reason is that in general there are only a few papers on the joint evolution of morphology and controller, the majority of work in evolutionary robotics considers the evolution of brains within a fixed body. Furthermore, the computational costs can be prohibitive, or at least discouraging. To illustrate the severity of this issue let us note that the infant learning process will need to generate several new brains and test them all in the given body. This means several additional fitness evaluations^1^ for each newborn robot. Giving newborn robots a learning budget of *N* new controllers to be generated and tested during the Infancy stage implies that the total number of fitness evaluations will be *N* times higher than evolving without learning. Of course, an appropriate value for *N* depends on the system and the application at hand, but a few hundred or a few thousand learning trials seem common in the literature.

The main goal of this research is to investigate the effects of learning in morphologically evolving robots. To this end, we set up a system where (simulated) modular robots can reproduce and create offspring that inherit the parents’ morphologies and controllers. Thus, in our system bodies and brains are both inheritable and therefore evolvable. Additionally, we make the brains not only evolvable, but also learnable. We achieve this by implementing a generic learning algorithm to optimize the inherited brain on any possible robot body.

This system is then tested by giving it a problem to solve, namely, the task of targeted locomotion. This task will be interfaced to the evolutionary system by an appropriate fitness function that quantifies a robots ability to move towards a pre-specified target. Applying mate selection and survivor selection mechanisms, which are based on this notion of fitness, drive evolution towards robots whose body and brain are well-suited for the given task. In order to investigate the effects of learning, we run the robot evolution process in two versions, namely, one purely evolutionary and one where evolution and learning are combined. In the first one, the brain of a robot child is inherited from its parents and its fitness is established directly after “birth”. In the second one, the inherited brain of a robot child is the starting point of a learning algorithm and the fitness of the robot will be based on its performance using the learned brain.

Based on these experiments, we address the issues of efficacy, efficiency, and compare the evolved morphologies. Regarding efficacy and efficiency, the main question is how the two algorithm variants compare in terms of the highest achieved performance and the time needed to achieve the best performance. Our underlying hypothesis is that learning forms a relatively fast and cheap way of reducing the number of slow and expensive evolutionary steps in a real-world application. To be specific, let us distinguish learning trials and evolutionary trials. An evolutionary trial equals to producing, activating and testing a new robot, including the manufacturing of its body, while a learning trial means producing, activating and testing a new controller in a given robot. Because controllers are digital entities (i.e., lines of code), learning trials require much less time, effort and material resources than evolutionary trials (Eiben, 2021). For instance, if the time of manufacturing a new robot is 12 h, the time needed for computing and installing a new controller to be tried is 10 s and the duration of one fitness evaluation is 1 minute, then 1,000 evolutionary trials need approximately 501 days, while 1,000 learning trials cost less than 1 day. Our corresponding research questions are the following.

**Research Question 1:** How much “savings” (in terms of evolutionary trials) can we achieve by adding infant learning as defined by the Triangle of Life methodology?

The answer to this question will indicate whether it is better to spend all allowable fitness evaluations on evolutionary trials (generating and testing a new robot consisting of a new body and a new brain) or should they be divided between evolutionary trials and learning trials (generating and testing a new brain in an existing body). Concerning the morphologies, we will consider quantifiable morphological traits, e.g., size, symmetry, and compare the trends during the evolutionary process as well as the morphologies in the final populations.

**Research Question 2:** Will the addition of learning lead to differently evolved morphologies?

Intuitively, one may expect no differences because learning is a process that only affects the brains, not the bodies. However, in the precursor of our current study, it has been found that adding learning can lead to different morphologies (Miras et al., 2020).

Additionally, we look into the robots’ ability to learn, specifically we investigate how this changes over the course of evolution. To quantify the learning potential of a given robot we use the *learning delta*, the performance difference between the robot right after birth with its inherited brain and robot after the learning stage using the learned brain. This notion reflects the ability of a given morphology to gain high performance through learning.

**Research Question 3:** How does the learning delta evolve over consecutive generations?

We consider the notion of the *learning delta* defined as the performance difference between the inherited and the learned brain, and find that it is growing throughout the evolutionary process.

## 2 Related Work

In the field of Evolutionary Robotics the majority of studies consider the evolution of brains (controllers) within a fixed body (morphology). Research into systems where morphologies and controllers both evolve is scarce. This is not surprising, considering that the simultaneous evolution of morphologies and controllers implies two search spaces and the search space for the brain changes with every new robot body produced. This makes research complex and challenging.

### 2.1 Evolvable Morphology

Similar research has been done recently with evolvable morphologies (Cheney et al., 2014; Lipson et al., 2016; Nygaard et al., 2017; Cheney et al., 2018; Nygaard et al., 2018; Wang et al., 2019; Liao et al., 2019; De Carlo et al., 2020; Zhao et al., 2020; Goff et al., 2021; Gupta et al., 2021; Medvet et al., 2021; Pagliuca and Nolfi, 2021). Among all the studies, several approaches have been proposed to mitigate the body and brain mismatch effect on the population.

Cheney et al. (2018) implemented a so-called morphological innovation protection mechanism which allows additional optimization of the controller in a “newborn” body by using mutations of the inherited brain. Technically this is similar to the Infancy stage concept within the Triangle of Life (Eiben et al., 2013). However, there is an important difference: during the protected period a robot can produce offspring (is eligible for parent selection), but it cannot be removed from the population (is exempted from survivor selection), whereas in our infant learning stage the robot is exempted from both selection mechanisms, it cannot produce offspring and it cannot be removed. This reveals a different rationale. Infant learning in the Triangle of Life scheme protects the population from being contaminated by the (possibly inferior) genes of a new individual, morphological innovation protection mechanism protects a new individual from being wiped out by the (possibly superior) population members.

Similarly, De Carlo et al. (2020) implemented protection in the form of speciation within their NEAT algorithm. The preservation of diversity in the population allowed new morphologies to survive, thus reducing the effects of body-brain mismatch.

Nygaard et al. (2017) demonstrated improvements in their ER system by introducing two phases during evolution. The first phase consists of both controller and morphology evolution, while during the second phase only the controller evolves in a fixed body. The results showed that, without the second phase, morphology and controller evolution led to sub-optimal controllers which required additional fine-tuning.

Zhao et al. (2020) used a graph grammar to express possible arrangements of physical robot assemblies. Each robot design is expressed as a sequence of grammar rules. Then they used the Graph Heuristic Search method to find the top performing robots and their corresponding controllers. In Graph Heuristic Search, they explore the design space while simultaneously learning a heuristic function used to guide the morphology search phase.

The work of Gupta et al. (2021) uses evolution for the robot bodies and reinforcement learning to optimize the controllers for the simulated robots. They introduced an asynchronous parallel evolution algorithm to reduce the computational budget by removing the typical generational aspect of an EA.

Goff et al. (2021) proposed the use of an external archive to store previously discovered controllers which are the highest-performing controllers found for the type. Then they provide learning on these controllers.

Combining evolution and learning in morphologically evolvable modular robots has been done successfully by Miras et al. (2020). Their study was the first to observe the evolution of the learning delta and they provided interesting evidence that a system change affecting the brains (the addition of learning) can cause measurable effects regarding the evolved bodies. They considered gait learning as the task and the representation of morphologies was based on L-systems. Here, we consider targeted locomotion as the task that is more complex and more relevant for practical applications than gait learning. Moreover, we use CPPNs as the genetic representation, and we apply a different learning algorithm than the one in (Miras et al., 2020).

### 2.2 Controller Learning Algorithms

Regarding the controller learning algorithms, there are many recent papers applying learning algorithms to the brains of robots with fixed bodies in order to produce optimal brains (Schembri et al., 2007; Ruud et al., 2017; Luck et al., 2019; Jelisavcic et al., 2019; Lan et al., 2020;; Schaff et al., 2019; Le Goff et al., 2020; van Diggelen et al., 2021; Nordmoen et al., 2021), naming only a few.

A comparison of three learning algorithms in modular robots is performed in (van Diggelen et al., 2021), where *Evolutionary Strategies*, *Bayesian Optimization* and *Reversible Differential Evolution* (RevDE) (Tomczak et al., 2020) are tested. The study does not use learning within an evolutionary system, rather it compares these algorithms as learners on a test suite of different morphologies. The outcomes indicate that the shape of the fitness landscape in Evolutionary strategies hints to a possible bias for morphologies with many joints. This could be an unwanted property for the implementation of lifetime learning because we want an algorithm that can work consistently on different kinds of morphologies. Bayesian Optimization is good at sample efficiency, however it requires much more time compared to the other two methods due to the higher time-complexity. The best performing algorithm in this comparison was RevDE. However, by its design, RevDE triples the size of the original population, thus, the evaluation step comes with an extra computational cost. Therefore, we use an advanced version of RevDE to alleviate this issue in this paper. This algorithm is introduced in the Algorithm section.

## 3 Experiment Setup

The experiments have been carried out in Revolve (https://github.com/ci-group/revolve), a Gazebo-based simulator which enables us to test parts of the system as well as to set an entire environment for the complete evolutionary process. All experiments were performed using an infinite plane environment to avoid any extra complexity. We ran two experiments: experiment 1 works by running evolution alone. In this system, controllers are inheritable and the controller of the offspring is produced by applying crossover and mutation to the controllers of the parents. We refer to this experiment as Evolution-Only throughout the paper. In experiment 2, controllers are not only evolvable, but also learnable. In these experiments, the controller of the offspring is produced by the learning algorithm that starts with the inherited brain. We refer to this experiment as Evolution + Learning throughout the paper.

### 3.1 Robot Morphology (Body)

#### 3.1.1 Phenotype of Body

**FIGURE 1:**
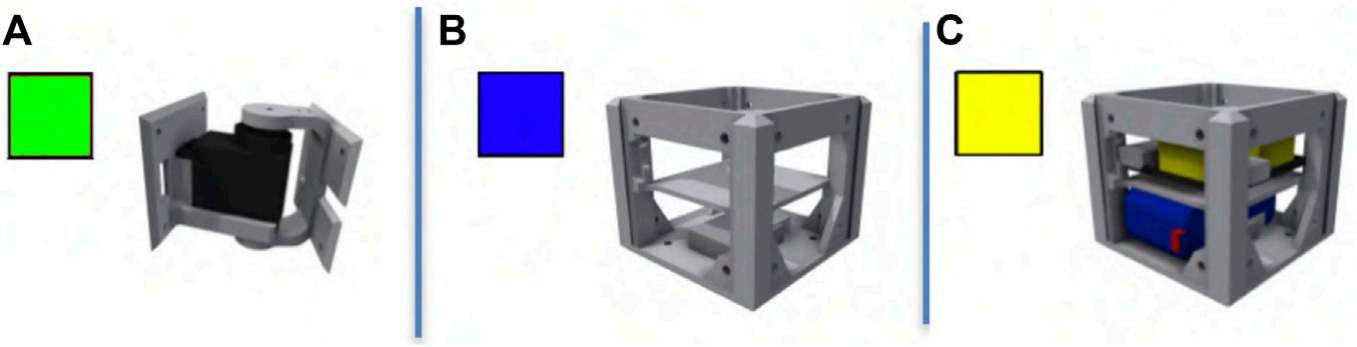
Modules of robots: The active hinge component **(A)** is a joint moved by a servomotor with attachment slots on both ends; The brick component **(B)** is a smaller cube with attachment slots on its lateral sides; The core component **(C)** is a larger brick component that holds a controller board and a battery.

The robots in Revolve are based on the RoboGen framework (Auerbach et al., 2014). We use a subset of RoboGen’s 3D-printable components: a morphology consists of one core component, one or more brick components, and one or more active hinges (see Figure 1). The phenotype follows a tree-structure, with the core module being the root node from which further components branch out. Child modules can be rotated 90° when connected to their parent, making 3D morphologies possible. The resulting bodies are suitable for 3D printing.

#### 3.1.2 Genotype of Body

The bodies are encoded in a Compositional Pattern Producing Network (CPPN) which was introduced in (Stanley, 2007). It has been demonstrated that with this encoding it is possible to evolve complex patterns for a variety of tasks (Clune et al., 2009), (Haasdijk et al., 2010), (Clune and Lipson, 2011), (Auerbach and Bongard, 2014), (Goff et al., 2021)].

The structure of the CPPN has four inputs and five outputs. The first three inputs are the x, y, and z coordinates of a component, and the fourth input is the distance from that component to the core component in the three structure. The first three outputs are the probabilities of the modules to be a brick, a joint, or empty space, and the last two outputs are the probabilities of the module to be rotated 0 or 90°. For both module type and rotation the output with the highest probability is always chosen.

The body’s genotype to phenotype decoder operates as follows: The core component is generated at the origin. We move outwards from the core component until there are no open sockets (breadth-first exploration), querying the CPPN network to determine the type and rotation of each module. Additionally, we stop when ten modules have been created. The coordinates of each module are integers; a module attached to the front of the core module will have coordinates (0,1,0). If a module would be placed on a location already occupied by a previous module, the module is simply not placed and the branch ends there.

### 3.2 Robot Controller (Brain)

#### 3.2.1 Phenotype of a Brain

We use Central Pattern Generators (CPGs)-based controllers (Ijspeert et al., 2007) to drive the modular robots. CPGs are biological neural circuits that produce rhythmic outputs in the absence of rhythmic input (Bucher et al., 2015). Earlier research has proven that this approach can produce stable and well performing gaits on both non-modular robots (Sproewitz et al., 2008; Christensen et al., 2013) and modular robots (Kamimura et al., 2005; Sproewitz et al., 2008; Pouya et al., 2010; Luo et al., 2021).

**FIGURE 2:**
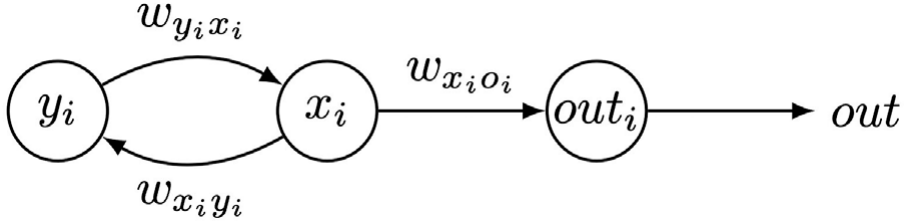
A single CPG. *i* denotes the specific joint that is associated with this CPG. $w_{x_{i}y_{i}}$ , $w_{y_{i}x_{i}}$ , and $w_{x_{i}o_{i}}$ denote the weights of the connections between the neurons, and *out* is the activation value of *out*_*i*_-neuron that controls the servo in a joint.

In this study, the controllers are optimized for learning targeted locomotion. Each robot joint is associated with a CPG that is defined by three neurons: an *x*_*i*_-neuron, a *y*_*i*_-neuron, and an *out*_*i*_-neuron that are recursively connected as shown in Figure 2.

The change of the *x*_*i*_ and *y*_*i*_ neurons’ states with respect to time is calculated by multiplying the activation value of the opposite neuron with a weight. To reduce the search space, we define $w_{x_{i}y_{i}}$ to be $-w_{y_{i}x_{i}}$ and call their absolute value *w*_*i*_. The resulting activations of neurons *x* and *y* are periodic and bounded. The initial states of all *x* and *y* neurons are set to $\frac{\sqrt{2}}{2}$ because this leads to a sine wave with amplitude 1, which matches the limited rotating angle of the joints.

$$\begin{array}{cc} & \dot{x_{i}}=w_{i}y_{i}\\ & \dot{y_{i}}=-w_{i}x_{i} \end{array}$$ (1)

To enable more complex output patterns, CPG connections between neighbouring joints are implemented. Two joints are said to be neigbours if their manhattan distance is less than or equal to two. *x* neurons depend on neighbouring *x* neurons in the same way as they depend on their *y* partner. Let *i* be the number of the joint, $N_{i}$ the set of indices of joints neighbouring joint *i*, and *w*_*ij*_ the weight between *x*_*i*_ and *x*_*j*_. Again, *w*_*ji*_ is set to be − *w*_*ij*_. The extended system of differential equations is then:

$$\begin{array}{cc} & {\dot{x}}_{i}=w_{i}y_{i}+\underset{j\in N_{i}}{\sum }w_{x_{j}x_{i}}x_{j}\\ & {\dot{y}}_{i}=w_{i}x_{i} \end{array}$$ (2)

Because of this addition, *x* neurons are no longer bounded between [ − 1, 1]. To achieve this binding, we use a variant of the sigmoid function, the hyperbolic tangent function (tanh), as the activation function of *out*_*i*_-neurons.

$$out_{\left(i,t\right)}\left(x_{\left(i,t\right)}\right)=\frac{2}{1+e^{-2x_{\left(i,t\right)}}}-1$$ (3)

#### 3.2.2 Genotype of a Brain

The structure of the brain largely depends on the body; only the weights between neurons can vary. Similarly to the morphology, we use CPPN as the genetic representation for the robot brain.

As each CPG corresponds to a joint, they have an associated three-dimensional position. The CPPN has six inputs: the coordinates of a CPG and the coordinates of another CPG connected to it. The output of the network is the weight between these CPGs. When querying for the weight between an *x* and a *y* neuron, the two input positions are both set to the same CPG coordinates. Although there are two weights between each two connected neurons, these are set to be the negative of each other. Because of this, the CPPN is only evaluated once, using coordinates of the neuron with the lowest index as the first three inputs.

#### 3.2.3 Steering Policy

In order to steer the modular robots, an additional steering policy is introduced. When the robot needs to turn right, joints on the right are slowed down, and visa versa. This does not lead to the correct steering behaviour for every robot, but we expect that emerging robots can use this policy successfully.

The magnitude of slowing down, *g*(*θ*), is derived from *θ*, the error angle between the target direction and the current direction. *θ* < 0 means that the target is on the left and *θ* > 0 means that the target is on the right. The target direction is calculated using the current absolute coordinates of the robot and the coordinates of the target point.

$$g\left(\theta \right)={\left(\frac{\pi -|\theta |}{\pi }\right)}^{n}$$ (4)

*n* is a parameter that determines how strongly the joints slow down. In this experiment we choose *n* = 7 based on manual fine-tuning.

Joints on the left side of the robot are controlled using the following formula:

$$signal=\left\{\begin{array}{ccc} & g\left(\theta \right)\cdot out & if\;\theta <0\\ & out & if\;\theta \geq 0 \end{array}\right.$$ (5)

Analogously, for joints on right side:

$$signal=\left\{\begin{array}{ccc} & out & if\;\theta <0\\ & g\left(\theta \right)\cdot out & if\;\theta \geq 0 \end{array}\right.$$ (6)

**FIGURE 3:**
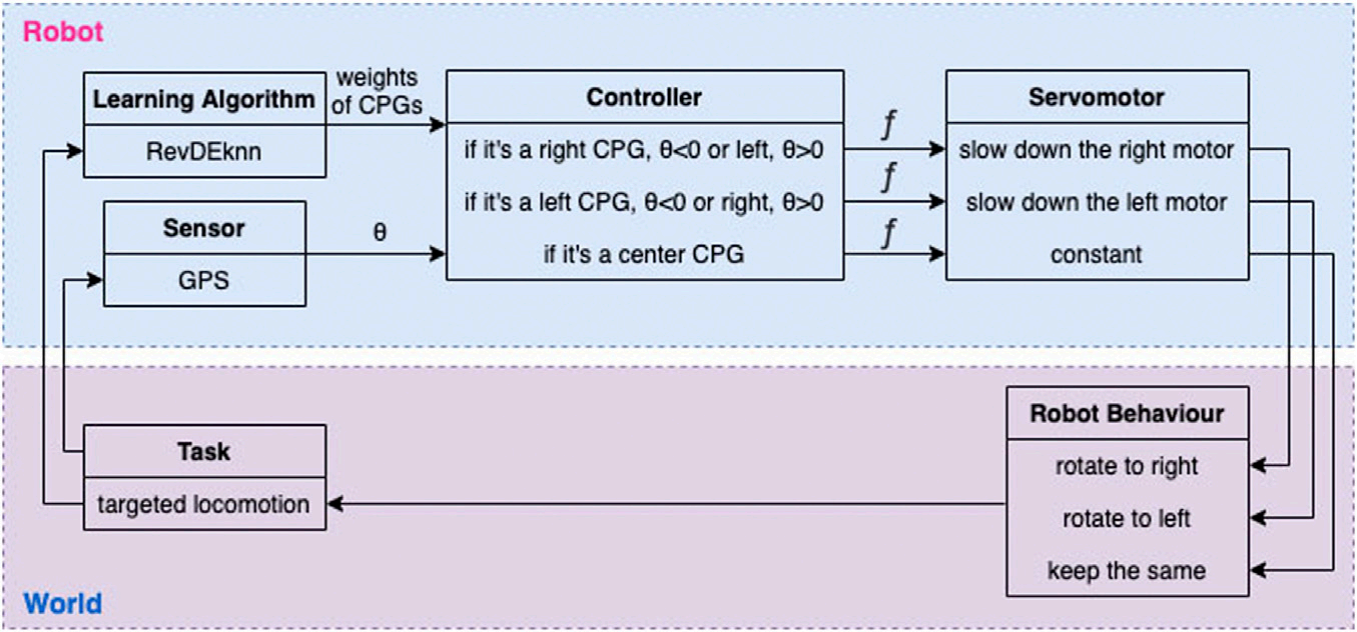
The overall architecture of how steering affects the joints. Error angle *θ* is calculated using the coordinates of the robot, its current heading, and its target. Function *f* (Eq. 5 & 6), which uses the output of the CPGs and *θ*, is then used to calculate the final signal going to the joints.

See Eq. 3 for the meaning of “out”. Figure 3 shows an overview of the complete control architecture.

### 3.3 Algorithms

We choose to use an evolutionary algorithm as the learning method to find good brain configurations for a given robot body. Previous work demonstrated that RevDE (Tomczak et al., 2020), an altered version of Differential Evolution, performs well in our type of modular robots (van Diggelen et al., 2021). However, the method used there increases the computational costs of running the simulator by tripling the population. Here, we follow the idea of introducing a surrogate model to overcome this issue (Weglarz-Tomczak et al., 2021). The model uses the K-Nearest-Neighbor (K-NN) regressor to approximate the fitness values of the new candidates, then select the N most promising points.

The new method, called RevDEknn, works as follows (Weglarz-Tomczak et al., 2021):

1. Initialize a population with X samples (*n*-dimensional vectors).
2. Assess all X samples.
3. Apply the reversible differential mutation operator and the uniform crossover operator.
     The reversible differential mutation operator: Three new candidates are generated by randomly picking a triplet from the population (*x*_*i*_, *x*_*j*_, *x*_*k*_) ∈ *X*, then all three individuals are perturbed by adding a scaled difference in the following manner:

$$\begin{array}{cc} y_{1} & =x_{i}+F\left(x_{j}-x_{k}\right)\\ y_{2} & =x_{j}+F\left(x_{k}-y_{1}\right)\\ y_{3} & =x_{k}+F\left(y_{1}-y_{2}\right) \end{array}$$ (7)

where *F* ∈ *R*_+_ is the scaling factor.

     New candidates *y*_1_ and *y*_2_ could be further used to calculate perturbations using points outside the population. This approach does not follow a typical construction of an EA where only evaluated candidates are mutated. Further, we can express (7) as a linear transformation using matrix notation by introducing matrices as follows:

$$\left[\begin{array}{c} y_{1}\\ y_{2}\\ y_{3} \end{array}\right]=\underset{=R}{\underset{︸}{\left[\begin{array}{ccc} 1 & F & -F\\ -F & 1-F^{2} & F+F^{2}\\ F+F^{2} & -F+F^{2}+F^{3} & 1-2F^{2}-F^{3} \end{array}\right]}}\left[\begin{array}{c} x_{1}\\ x_{2}\\ x_{3} \end{array}\right]$$ (8)

In order to obtain the matrix R, we need to plug *y*_1_ into the second and third equation in Eq. 7, and then *y*_2_ into the last equation in Eq. 7. As a result, we obtain *N* = 3*X* new candidate solutions and the linear transformation *R* is reversible.

     The uniform crossover operator: The authors of (Storn, 1997) proposed to sample a binary mask *m* ∈ {0,1}^*D*^ according to the Bernoulli distribution with probability *p* shared across *D* dimensions, and calculate the final candidate according to the following formula:

$$v=m⊙y_{n}+\left(1-m\right)⊙x_{n}.$$ (9)

Following general recommendations in literature (Pedersen, 2010) to obtain stable exploration behaviour, the crossover probability *p* is fixed to a value of 0.9 and the scaling factor *F* is fixed to a value of 0.5.

4. Apply K-NN to predict the assessment value of new samples (X + N) based on the *K* closest previously seen samples. The K-NN regression model is a non-parametric model that stores all previously seen individuals with their evaluations, and the prediction of a new candidate solution is an average over the K closest previously seen individuals (Table 1). In this paper, we set *K* = 3.
5. Perform a selection over the population based on the prediction and select X samples.
6. Repeat from step (2).
7. Terminate when the maximum number of iterations is reached.

**TABLE 1:** Parameters.

RevDEknn	Value	Description
*X*	10	Initial sample size
*F*	0.5	Scaling factor
*P*	0.9	Crossover probability
*K*	3	Number of Nearest-Neighbors
*G*	10	Number of iterations

**FIGURE 4:**
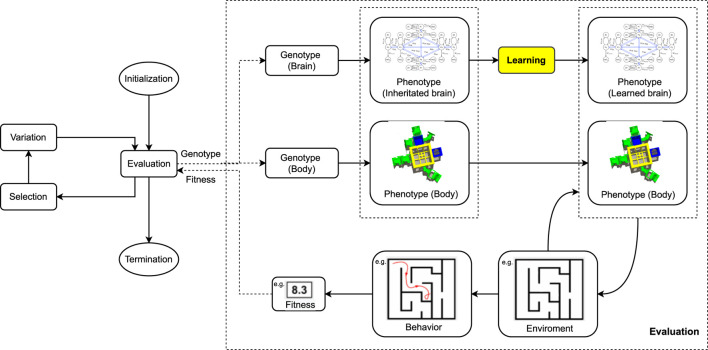
Evolution + Learning Framework. This is a general framework for optimizing robots via two interacting adaptive processes. The evolutionary loop (left) optimizes robot morphologies and controllers simultaneously using genotypes that encode both morphologies and controllers. The learning loop (yellow box inside the Evaluation step of the evolutionary loop) optimizes the controller for a given morphology. Note that in general the fitness measure used within the evolutionary loop need not be the same as the quality measure used inside the learning method, cf. Algorithm 1.

As explained above, we apply RevDEknn here as a learning method for “newborn” robots. In particular, it will be used to optimize the weights of the CPGs of our modular robots for the task of targeted locomotion during the Infancy stage. The complete integrated process of evolution and learning is illustrated in Figure 4, while Algorithm 1 displays the pseudocode. The initial population of *X* = 10 weight vectors for RevDEknn is created by using the inherited brain of the given robot. Specifically, the values of the inherited weight vector are altered by adding Gaussian noise to create mutant vectors and the initial population consists of nine such mutants and the vector with the inherited weights.

The code for carrying out the experiments is available online: https://bit.ly/3t99vjC.

Algorithm 1 Evolution + Learning

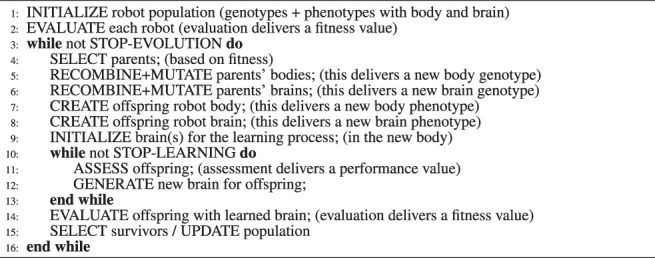

Note that for the sake of generality, we distinguish two types of quality testing depending on the context, evolution or learning. Within the evolutionary cycle (line 2 and line 14) a test is called an evaluation and it delivers a fitness value. Inside the learning cycle (line 11) a test is called an assessment and it delivers a performance value. This distinction reflects that in general the notion of fitness can be different from the task performance, perhaps more complex involving more tasks, other behavioral traits not related to any task, or even morphological properties.

### 3.4 Fitness Function

CPG controllers that are learning targeted locomotion in modular robots with evolvable morphologies pose a black-box optimization problem. We therefore need to formulate a fitness function to reach our objective. In our system, the fitness function is not only used to evaluate the performance of robots but also serves as the guiding metric of learning controllers.

We define a fitness function for targeted locomotion that fulfills the following three objectives:

1. Minimize deviation with respect to the given target point.
2. Maximize speed (by minimizing the length of the trajectory).
3. Increase robustness (by using not one, but more target points for testing).

**FIGURE 5:**
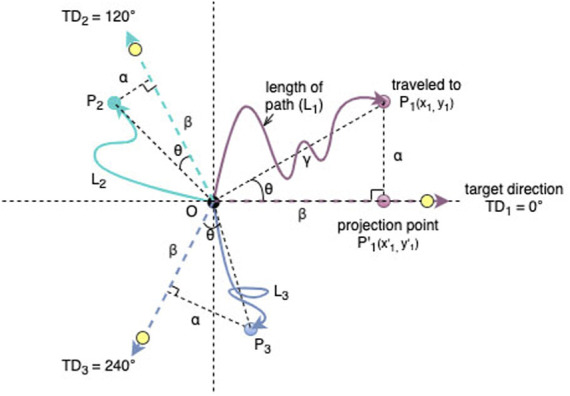
Illustration of the fitness function. We use 3 target points (yellow dots) to mark 3 target directions *TD*_1_=0°(purple dotted line), *TD*_2_=120°(green dotted line) and *TD*_3_=240°(blue dotted line). Point O is the starting position of the robot, *p*_1_ (*x*_1_, *y*_2_) is an example of the end position of a robot learning target direction *T*_1_. The purple line *L*_1_ is the actual travel path between (0,0) and *P*_1_. The point $P_{1}^{\prime }\left(x_{1}^{\prime },y_{1}^{\prime }\right)$ is the projection of *P*_1_ on the target direction *T*_1_. Same with the actual travel path *L*_2_ and *L*_3_ for *T*_2_ and *T*_3_ respectively.

The scenario of each robot’s fitness evaluations with 3 target points to mark 3 target directions in our experiments is illustrated in Figure 5.

The elements we denote are:

1. *α*, the distance between the projection point and the end point;
2. *β*, the distance between the projection point and the starting point *O* (0, 0);
3. *γ*, the distance between the starting point and the end point;
4. *θ*, the deviation angle between the actual direction of the robot locomotion and the target direction at time *T*_50_;
5. L , the path length of an individual’s trajectory.

The data we can collect from the simulator is as follows:

1. The coordinates of the core component of the robot at the start of the simulation (timestamp *T*_0_) is approximate to O (0,0).
2. The coordinates of the robot, sampled during simulation at 5 Hz, allowing us to plot and approximate the length of the paths ( $L_{1}$ , $L_{2}$ , $L_{3}$ ).
3. The end points *P*_1_ (*x*_1_, *y*_1_), *P*_2_ (*x*_2_, *y*_2_), *P*_3_ (*x*_3_, *y*_3_) are the coordinates of the core component of the robot at the end of the simulation (timestamp *T*_50_) with 3 target directions.
4. The orientation of the robot in every 0.2 s incremental timestamp plus the angle *δ* at timestamp *T*_50_ as calculated by using the end coordinates.

To achieve the first objective, we have to set multiple directions for training. In our fitness design, we select 3 fixed target directions equally distributed as *TD*_1_ = 0°, *TD*_2_ = 120°and *TD*_3_ = 240°. In this way, we avoid having robots whose morphologies are suited for one direction only (which coincidentally matches the randomly selected direction).

To achieve the second objective, we have to minimize the angle *θ* which can be calculated as follows:

$$\theta =\left\{\begin{array}{cc} & 2\pi -\left|\delta -TD\right|\;\;\;\left(\left|\delta -TD\right|>\pi \right)\\ & \left|\delta -TD\right|\;\;\;\;\;\;\left(\left|\delta -TD\right|\leq \pi \right) \end{array}\right.$$ (10)

Moreover, we also consider the deviation from the target direction by calculating the distance *α*. By knowing the coordinates of the end position *P*_*n*_(*x*_*n*_, *y*_*n*_), we can use Euclidean distance to the point of origin to first calculate *γ*:

$$\gamma =\sqrt{{\left(x_{n}\right)}^{2}+{\left(y_{n}\right)}^{2}}$$ (11)

Then we can calculate *α* as follows:

$$\alpha =\gamma \cdot \sin\left(\theta \right)$$ (12)

To have an efficient targeted locomotion, just moving in the right direction by achieving objective 1 and 2 is not enough, the robot should also move as fast as possible in the target direction which leads to the third objective. Given the same evaluation time, the higher speed means the longer distance travelled along the target direction. We calculate the distance travelled by the robot in the target direction by projecting the final position at timestamp *T*_50_ onto the target direction, e.g., the point $P_{1}^{\prime }\left(x_{1}^{\prime },y_{1}^{\prime }\right)$ is the projection point of *P*_1_ (*x*_1_, *y*_1_).

We can calculate the projected distance in the target direction as follows:

$$\beta =\mathrm{sign}\left(\gamma \cdot \cos\left(\theta \right)\right)$$ (13)

where sign = 1 if $\theta <\frac{\pi }{2}$ and sign = −1 otherwise. This results in the *β* being negative when the robot moves in the opposite direction.

Besides maximizing the projected distance, we also have to consider minimizing the length of the actual path of the robot’s trajectory to have a robot that walks a more efficient trajectory. We sum up all the ds values given the fixed evaluation time as the L .

In order to create a more robust fitness function, we introduce a penalty factor *ω* to fine-tune the result. *ω* is a constant scalar and in the experiments we set it to be 0.01.

The above results in the following fitness function:

$$F_{\left(\beta ,\alpha ,\theta ,L\right)}=\frac{\left|\beta \right|}{L+\varepsilon }\cdot \left(\frac{\beta }{\theta +1}-\omega \cdot \alpha \right)$$ (8)

where *ɛ* is an infinitesimal constant (set to 10^–10^ in the experiments). *β* is divided by *θ* to further reward a small deviation angle (which is increased with 1 to prevent from dividing by 0). The function is maximised when *β* is high, and L , *θ*, and *α* equal zero. In case of zero deviation from the target direction, $F_{\left(\beta ,\alpha ,\theta ,L\right)}=\beta$ .

To prevent passive individuals from taking over the population (potential local optimum), we set the fitness to zero for organisms that move less than 10 cm towards the target direction. When it comes to the implementation of the code, the target points *P*_*i*_ that define the target directions *TD*_*i*_ are placed 10 m from the point of origin (0,0).

### 3.5 Experiment Parameters

For the evolutionary loop, we use a variant of the well-known (*μ* + *λ*) selection mechanism with *μ* = 100 and *λ* = 50 to update the population. An initial population of 100 robots is randomly generated to form the first generation. In each generation 50 offspring are produced by selecting 50 pairs of parents through binary tournaments (with replacement) and creating one child per pair by crossover and mutation. To this end, we use the same mutation and crossover operators as in MultiNEAT (https://github.com/MultiNEAT/). The next generation is formed by the top 50 parents plus the 50 offspring. The evolutionary process is terminated after 30 generations.

In this paper, the fitness measure to drive evolution and the performance measure to drive learning are the same by design. Thus, we use the same test procedure, simulating one robot for 50 simulated seconds, for the evolutionary as well as the learning trials. For running the Evolution-Only algorithm we perform (50 +50×30) robots × 3 targeted directions = 4,650 fitness evaluations. In the Evolution + Learning algorithm we apply RevDEknn on each robot body resulting in 100 extra fitness evaluations (population size 10, multiplied by 10 generations). This implies (50+50×30) × 3 × 100 = 465, 000 fitness evaluations in total. To sum up, for running an Evolution-Only and an Evolution + Learning experiment we perform 469,650 evaluations which amounts to 469, 650, ×, 50/60/60 = 6, 523 h of simulated time. In practice, it takes about 13–14 days on one of our workstations. To get a robust assessment of the performance all the experiments are repeated 10 times independently. The experimental parameters we used in the experiments are described in Table 2.

**TABLE 2:** Main experiment parameters.

Parameters	Value	Description
Population size	100	Number of individuals per generation
Offspring size	50	Number of offspring produced per generation
Mutation	0.8	Probability of mutation for individuals
Crossover	0.8	Probability of crossover for individuals
Generations	30	Termination condition for each run
Learning trials	100	Number of the evaluations performed by RevDEknn on each robot
Evaluation time	50	Duration of the test period per fitness evaluation in seconds
Tournament size	2	Number of individuals used in the parent selection - (k-tournament)
*λ*/*μ*	0.5	The ratio used in the survivor selection - (*μ* + *λ*)
Repetitions	10	Number of repetitions per experiment

### 3.6 Performance Measures

To compare the two methods, we consider two generic performance indicators, *efficacy*, *efficiency*, and a special measure, the learning delta.

#### 3.6.1 Efficacy and Efficiency

We measure efficacy in two ways. First, we do it by averaging the maximum fitness over the 10 independent repetitions achieved at the end of the evolutionary process (30 generations). Since we consider targeted locomotion here, the quality is defined by the fitness value. As this measure can be sensitive to ‘luck’, we get more useful statistics by taking the average over 10 different runs. Second, another way to measure the quality of the solution is by giving the same computational budget and measure which method finds the best solution (highest fitness) faster (i.e., within a fewer number of iterations).

Efficiency indicates how much effort is needed to reach a given quality threshold (the fitness level). In this paper, we use the number of *Evaluations-to-Solution* to measure it.

#### 3.6.2 Descriptors

For quantitatively assessing morphological traits of the robots, we utilize the following set of morphological descriptors:

**The Absolute Size**: Total number of modules of a robot body. It is a sum of all the structural bricks, hinges and one core-component.

**Proportion:** The length-width ratio of the rectangular envelope around the morphology. It is defined as following:

$$P=\frac{p_{s}}{p_{l}}$$

where *p*_*s*_ is the shortest side of the morphology, and *p*_*l*_ is the longest side.

**The Number of Bricks**: The number of structural bricks in the morphology.

**The Relative Number of Limbs**: The number of extremities of a morphology relative to a practical limit. It is defined as following:

$$L=\left\{\begin{array}{cc} \frac{l}{l_{\max}}\; & \text{if}\;l_{\max}>0\\ 0\; & \text{otherwise} \end{array}\right.$$

$$L_{\text{max}}=\left\{\begin{array}{cc} \frac{2\cdot \left(m-6\right)}{3}+\left(m-6\right)\left(\mathrm{mod}3\right)+4\; & \text{if}\;l_{\max}>0\\ m-1\; & \text{otherwise} \end{array}\right.$$

where *m* is the total number of modules in the morphology, *l* is the number of modules which have only one face attached to another module (except for the core-component) and *l*_max_ is the maximum amount of modules with one face attached that a morphology with *m* modules could have, if containing the same amount of modules arranged in a different way.

**Symmetry**: The ratio between the horizontal and vertical symmetry given by the morphology.

**Branching**: The ratio between the total number of modules that have an attachment on all four possible lateral sides and the maximum number of modules that could have had an attachment on all sides given the morphology.

We use the above six morphological descriptors to capture relevant robot morphological traits, and quantify the correlations between controller and morphology search spaces.

For quantitatively assessing behavioural characteristics of the robots, we utilized the displacement velocity descriptor which is defined as the distance between the last and first recorded position of the robot divided by the amount of travelling time, ignoring the length of the path that was taken.

## 4 Experiment Results

### 4.1 Efficacy and Efficiency

**FIGURE 6:**
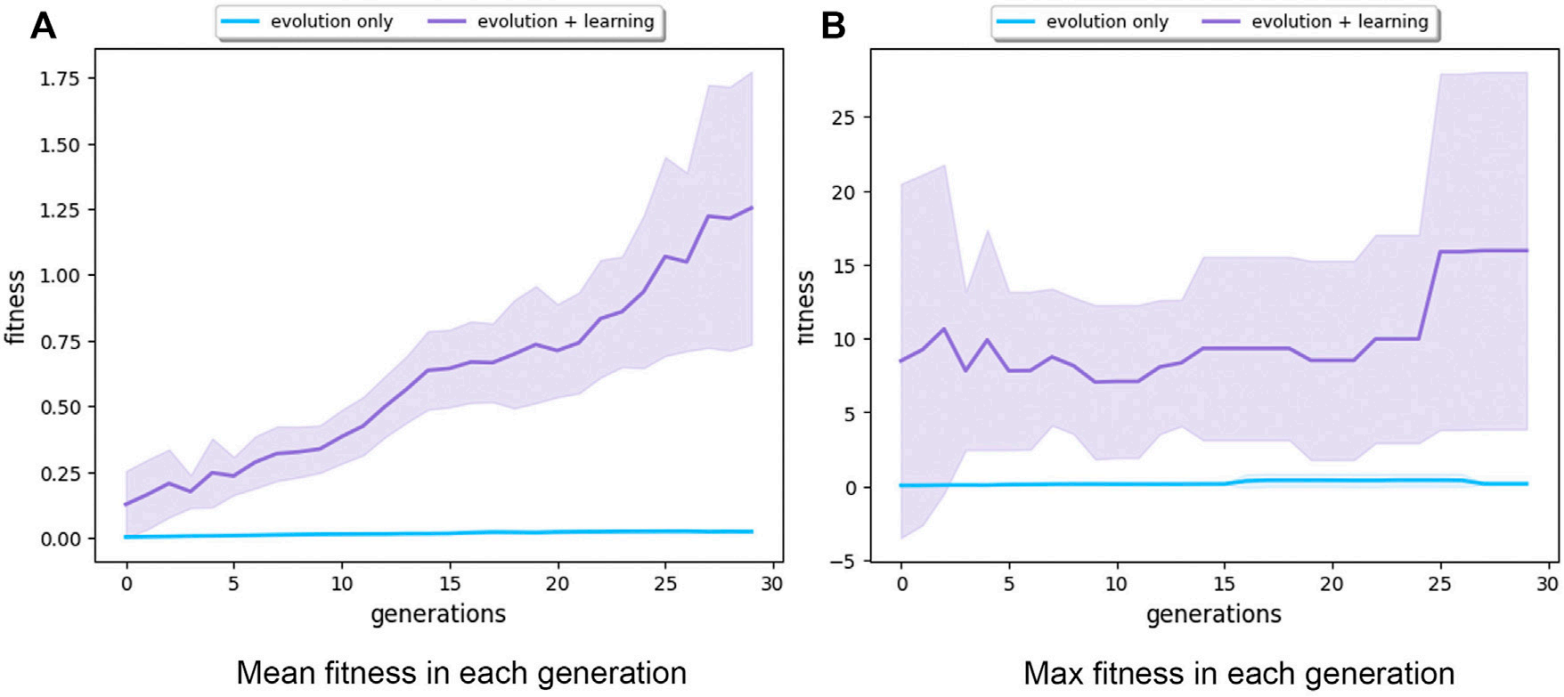
Subfigure **(A)** shows the mean fitness over 30 generations (averaged over 10 runs) for Evolution-Only in blue and Evolution + Learning in purple. The Evolution + Learning method already surpassed in its first generation the fitness levels that the Evolution-Only method achieved at the end of the evolutionary period. Subfigure **(B)** exhibits the maximum fitness in each generation (averaged over 10 runs). The shaded areas show the standard deviation. Note the different scales on the vertical axes.

Regarding efficacy, it is apparent that adding a life-time learning capacity to the evolutionary system increased the obtained fitness values, as depicted in Figure 6. It shows that at the first generation, Evolution + Learning had already significantly outperformed Evolution-Only at the end of the run (i.e., 30 generations). Further, we can observe that Evolution + Learning produced 100 robots and spent 30,000 evaluations in the first generation, while Evolution-Only produced 1,550 robots and executed 4,650 evaluations in those 30 generations. This may be confusing when comparing efficiency, but we argue that counting the number robots is more relevant here. The reason is that the time to produce a real robot (typically a few hours) is substantially higher than the time to run a fitness evaluation (less than 1 minute). From this perspective, the advantage of introducing learning is significant.

The dominating performance of Evolution + Learning could be explained in two ways: 1) the number of evaluations performed by Evolution + Learning is around 100 times higher than with Evolution-Only; 2) in Evolution + Learning, robots have time to fine-tune their controllers to the morphologies they were born with.

**FIGURE 7:**
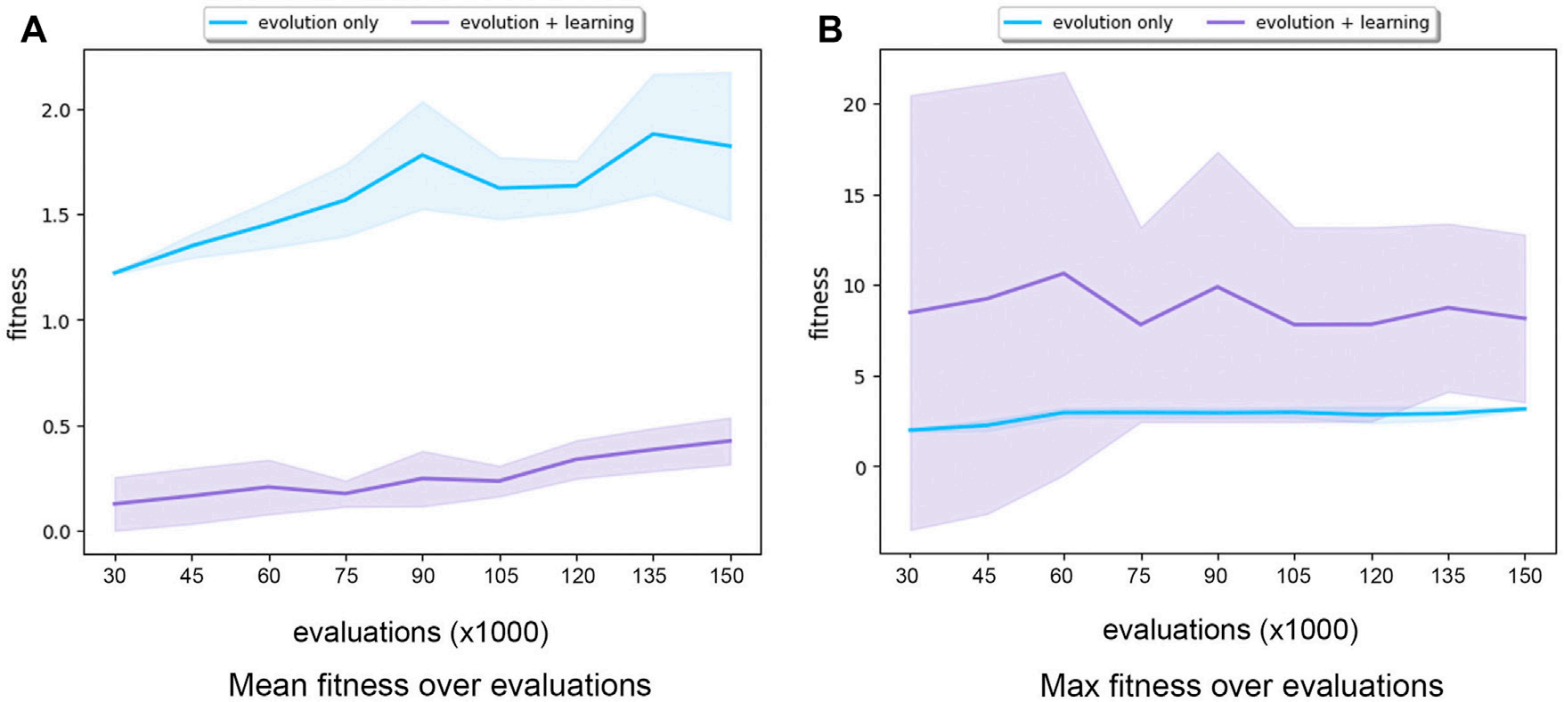
Subfigure **(A)** shows the mean fitness over 150,000 evaluations (averaged over 10 runs) for Evolution-Only in blue and Evolution+Learning in purple. Subfigure **(B)** shows the maximum fitness over 150,000 evaluations (averaged over 10 runs) for Evolution-Only in blue and Evolution+Learning in purple. The shaded areas show the standard deviation. Note the different scales on the vertical axes.

**FIGURE 8:**
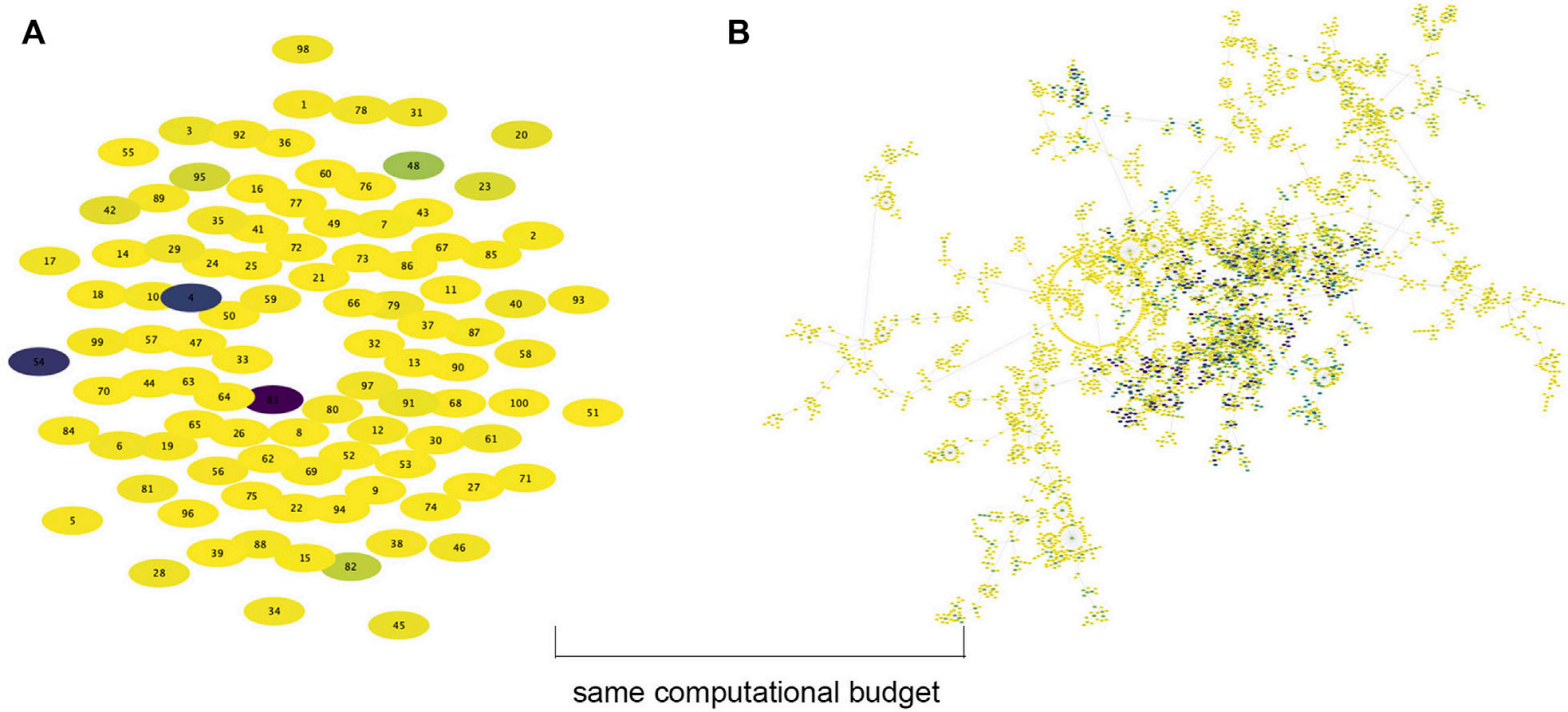
The genealogical trees of a single evolutionary run of both methods with the same computational budget: 30,000 evaluations. Each elliptical node represents a single robot. The node color indicates fitness (the darker the color the higher the fitness). The highest fitness found by Evolution + Learning is 31.90 (robot 83), whereas the highest fitness found by Evolution-Only is 1.92 at the end of 199 generations. **(A)** Evolution + Learning (generation 1). **(B)** Evolution Only (generation 199).

Figure 7 shows the mean and max fitness over the same number of evaluations. Evolution + Learning spent 150,000 evaluations created 500 robots at generation 9 while Evolution-Only created 50,000 robots at generation 999. Very interestingly we can see that the average mean of Evolution-Only is slightly higher than Evolution Learning while with regards to the max fitness, Evolution + Learning has much higher fitness and standard deviation. In other words, with learning, the robots with higher fitness and lower fitness differ more. This result is rather intriguing. Therefore, in order to get an insight into how the fitnesses of individual robots spread out in both methods, we plot the genealogical trees (see Figure 8).

Figure 8 shows the genealogical trees of a single evolutionary run of both methods with the same computational budget (evaluations = 30,000 and simulation time about 416.6 h). In this computing time regime, Evolution-Only evolved 199 generations while Evolution + Learning only generated the initial population of 100 without any offspring yet. In both subplots, each elliptical node represents a single robot. The node color (from light yellow to dark purple) reflects fitness, with darker nodes indicating higher fitness. The plot on the left shows the first generation of Evolution + Learning without any connections to descendants yet. The plot on the right shows 199 generations of Evolution-Only. The circle of nodes in the middle shows the initial population of 100. The nodes connected are their descendants. This tree demonstrates that founders with higher fitness intent to generate more lineages and higher fitness descendants. From both plots, we can see that although Evolution-Only generates many fit robots, the best solution (max fitness: robot ID 83 with fitness of 31.90) is found in Evolution + Learning, whereas the max fitness found by Evolution-Only is 1.92 at the end of 199 generations. In other words, if we want to achieve the target fitness level of 1.92, it takes Evolution-Only 30,000 evaluations, while it only takes Evolution + Learning less than 249 evaluations (83 robots ⋅ 3 target directions).

**FIGURE 9:**
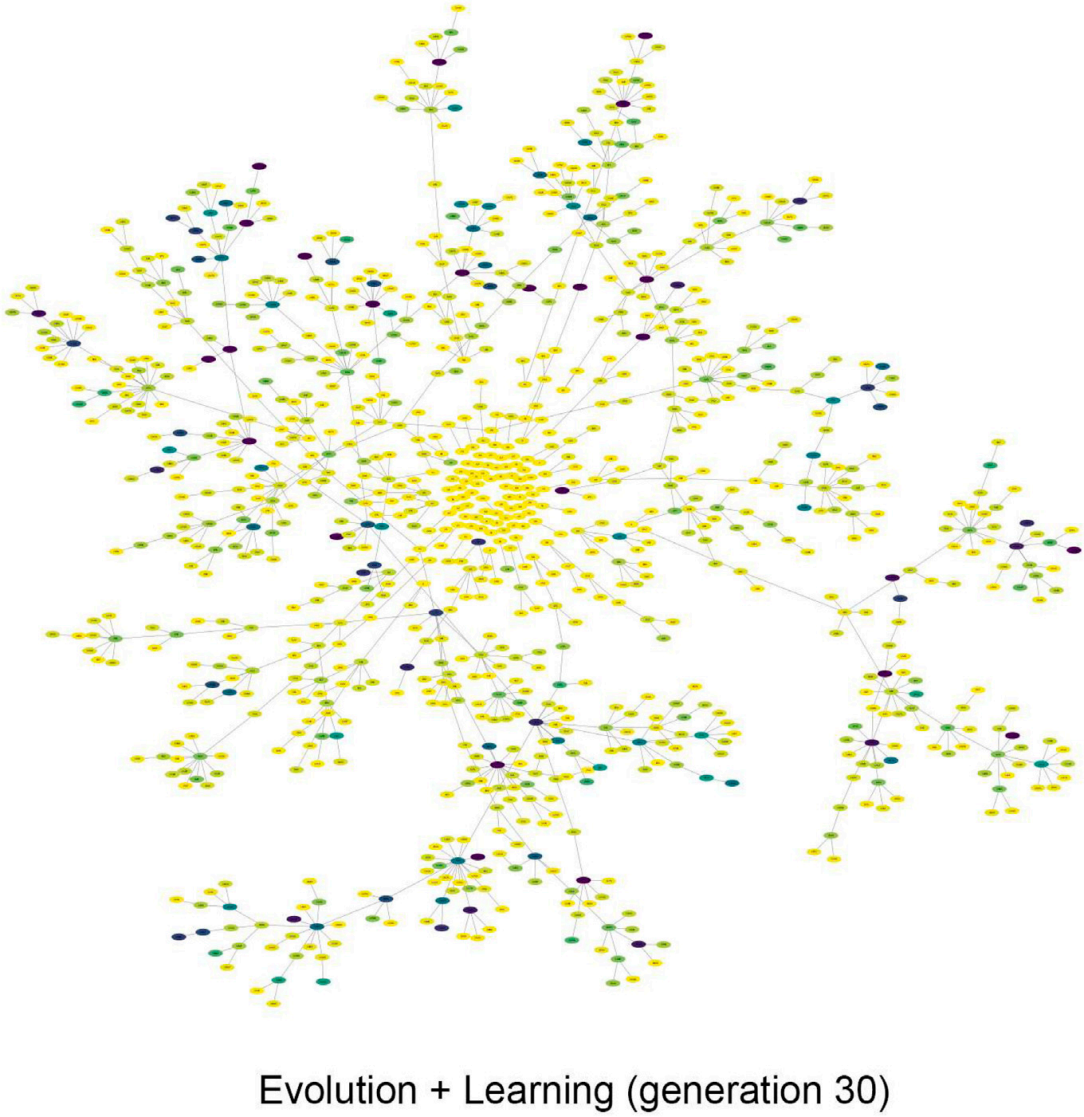
The genealogical tree of Evolution + Learning at generation 30. The descendants are spread out evenly across generations, as are the individuals with the highest fitness. It also demonstrates that descendants with high fitness can originate from ancestors with lower fitness (i.e., lighter nodes), rather than concentrating themselves in lineages that started with a high fitness in Evolution-Only as shown in Figures 8–b (‘fitness begets fitness’).

Figure 9 shows the genealogical tree of Evolution + Learning at the last generation. Similar to Figure 8, the round circle in the middle shows the initial population of 100, then it is developed in a circular layout. The darker nodes indicating the higher fitness. Compare to Figure 8. b, the descendants are more evenly spread out generation by generation, so do the max fitness individuals. It demonstrates that multiple lineages with descendants of high fitness can originate from founders with lower fitness (i.e. lighter nodes), rather than converging to one direction as shown in Figure 8. b.

### 4.2 Morphology

#### 4.2.1 Morphological Descriptors

In (Luo et al., 2021), a study utilizing the same robot framework but using L-system as genetic encoding, a strong selection pressure for robots with few limbs was observed. In particular one single, long limb, i.e., a snake-like morphology. In this paper, we use CPPN as genetic encoding and the morphological traits of robots from Evolution-Only are similar to (Luo et al., 2021), however we observe different morphological development trends in Evolution + Learning.

**FIGURE 10:**
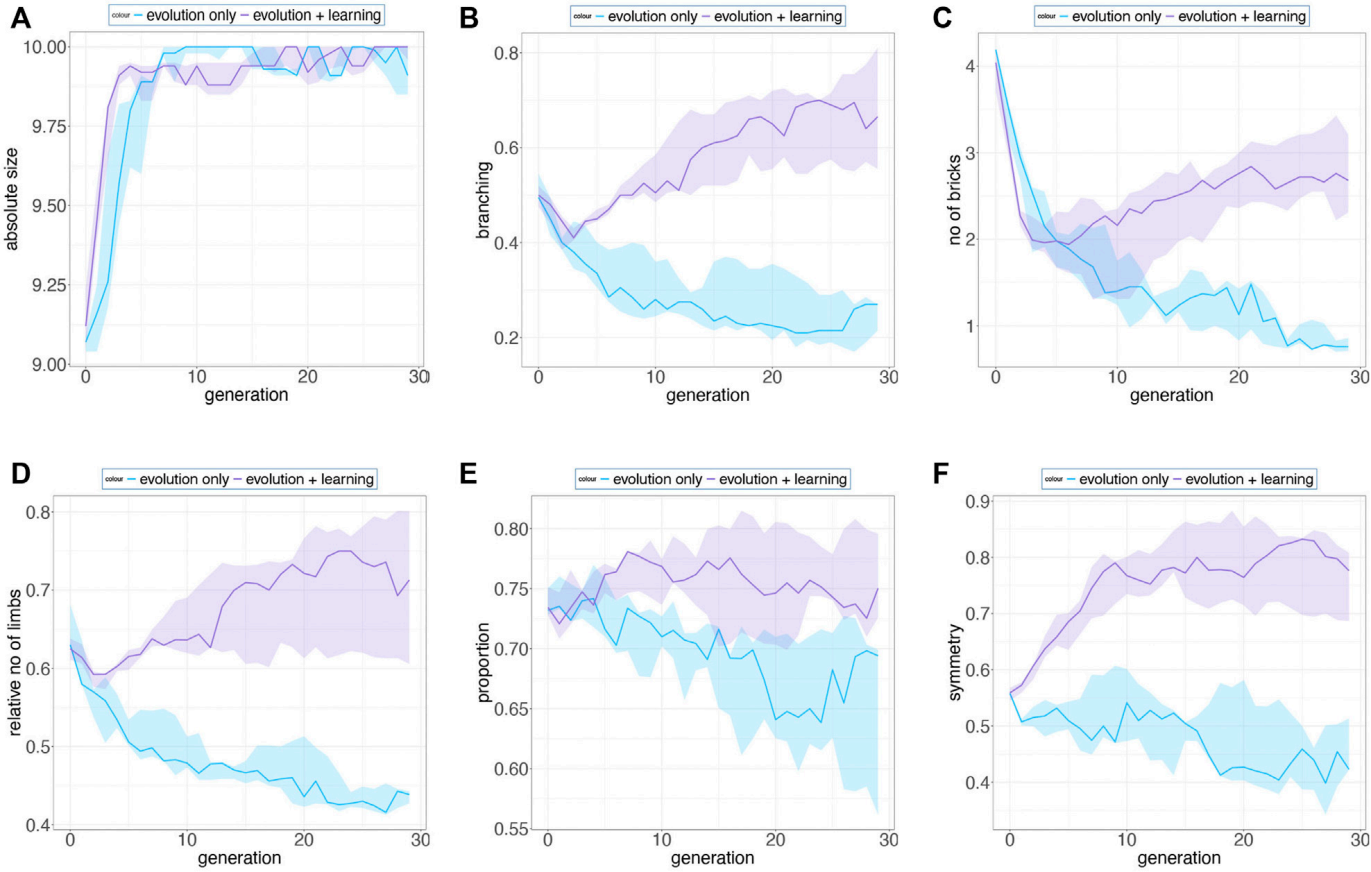
We selected 6 morphological traits that display a clear trend over generations and present the progression of their means averaged over 10 runs for the entire population. Shaded regions denote a 95% bootstrapped confidence interval. **(A)**, robots from both methods tend to develop bigger sizes over generations **(B) (D)**, branching parameter and the relative no. of limbs from Evolution + Learning are increasing along the evolutionary timeline, however Evolution-Only evolved in an opposite manner **(C) (F)**, Evolution + Learning robots tend to be more symmetric and have more bricks as they evolved compared to robots evolved in Evolution-Only **(E)**, proportions from both methods show slightly decreasing trends, and the ratio of width and length of the robot morphology in Evolution + Learning is slightly higher over generations.

We selected 6 morphological traits which display a clear trend over generations. In Figure 10, we see the progression of the mean of different morphological descriptors averaged over 10 runs for the entire population.

**FIGURE 11:**
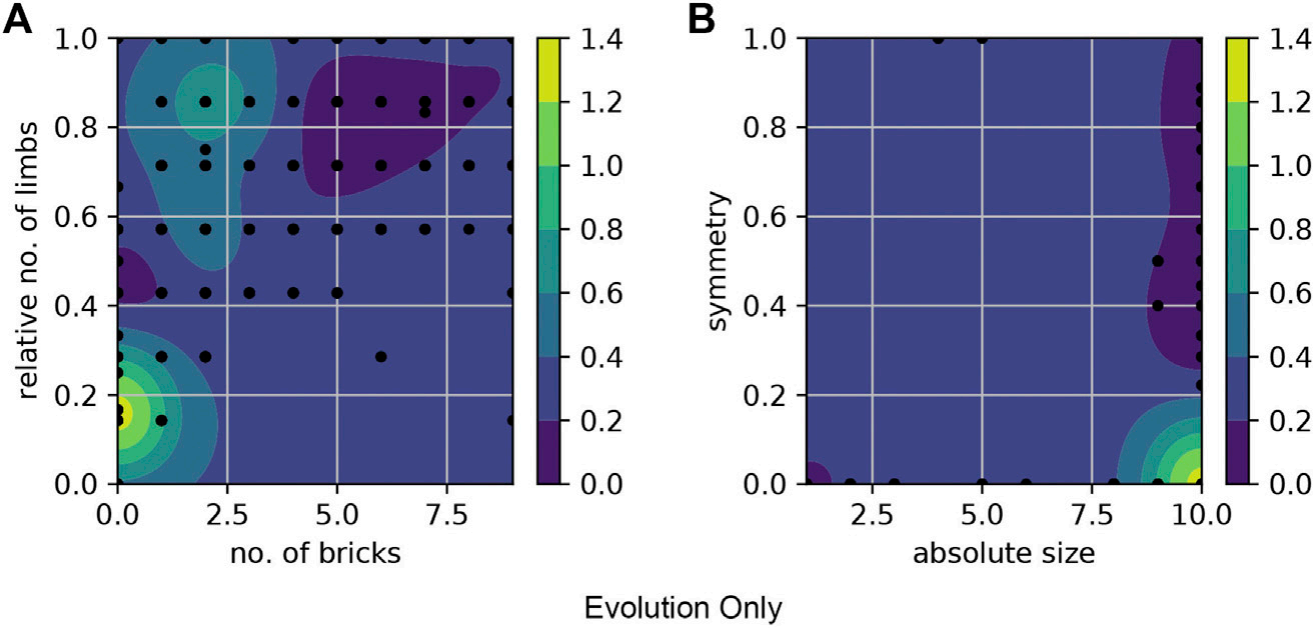
Fitness landscape of Evolution-Only using pairs of morphological measurements as coordinates. The color bars show the fitness level. **(A)** shows the fitness landscape of number of bricks over relative number of limbs, the robots with higher fitness values are in the morphological spaces of fewer bricks and limbs. **(B)** shows the fitness landscape of absolute size over symmetry. The robots with the highest fitness have a maximum absolute size of 10, however they are not symmetrical.

**FIGURE 12:**
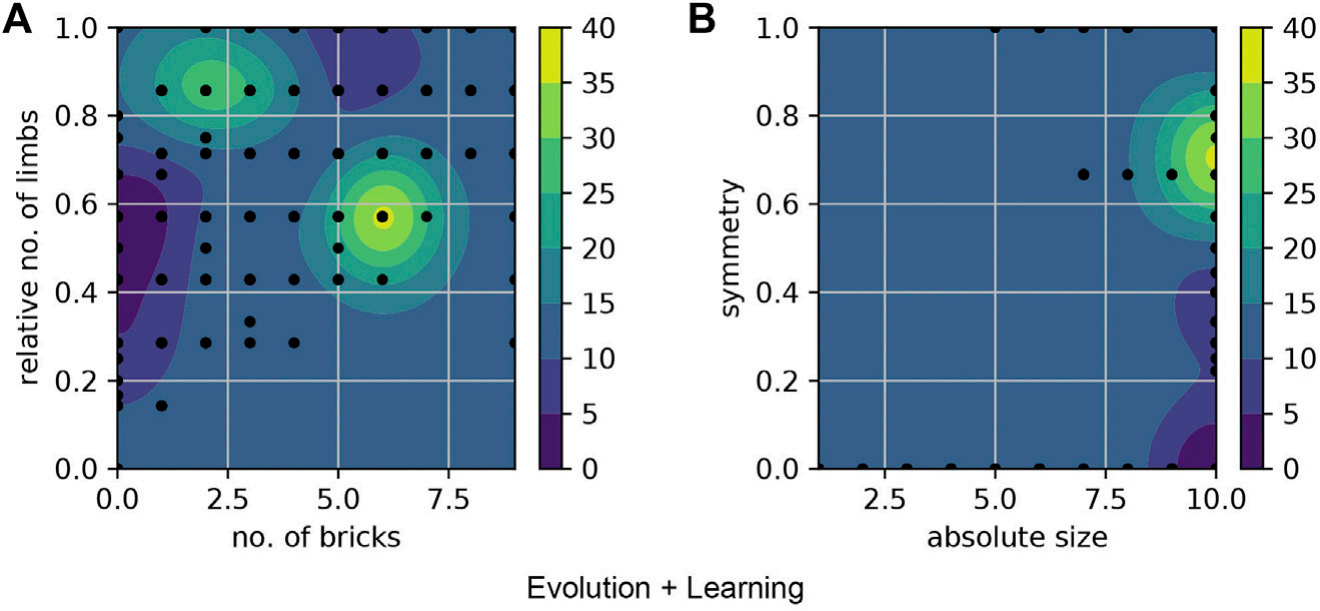
Fitness landscape of Evolution + Learning. **(A)** the robots with higher fitness are in the regions where the contour color is brighter (yellow). **(B)** shows the fitness landscape of absolute size over symmetry. The highest fitness robots are highly symmetrical.

We present the fitness landscape plots (Figure 11 and Figure 12) using pairs of morphological measurements as coordinates. Figure 11. a and Figure 12. a show the fitness landscape as a function of number of bricks over relative number of limbs of these two methods. In Evolution-Only, the robots with higher fitness are in the morphological spaces of fewer bricks and limbs, while in Evolution + Learning, in the high fitness region where the contour color is brighter and wider are the robots with relatively more bricks and limbs. Figure 11. b and Figure 12. b show the fitness landscape of absolute size over symmetry of these two methods. In both methods, the robots with the highest fitness have a maximum absolute size of 10, however with regards to the symmetry, the highest fitness robots from Evolution + Learning are highly symmetrical whereas the ones from Evolution-Only are not.

**FIGURE 13:**
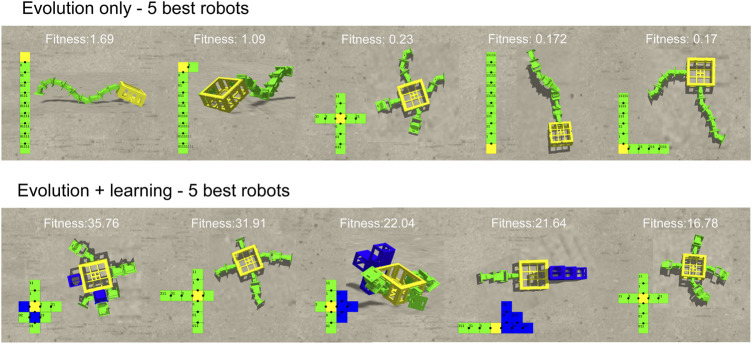
The 2D and 3D morphologies of 5 best robots from both methods. The best robots generated by Evolution + Only are all converged into the morphology type that only contain hinges and no more bricks while the best robots from Evolution + Learning are more diverse. From the 3D morphologies we can see that the robots from Evolution-Only are evolved with one layer while the robots from Evolution + Learning are developed in the horizontal dimension as well. With regards to the fitness, robots from Evolution-Only are suffering from local optimum. In these 10 robots, we observe a same morphology from both methods (cross-shape), however the robot from Evolution + Learning has a much higher fitness value, once again it shows the importance of matching body and brain.

In Figure 13, it shows the 2D and 3D morphologies of 5 best robots from both methods. The best robots generated by Evolution-Only are all converged into the morphology type that only contain hinges and no more bricks while the best robots from Evolution + Learning are more diverse. From the 3D morphologies we can see that the robots from Evolution-Only are evolved with one layer while the robots from Evolution + Learning are developed in the horizontal dimension as well. With regards to the fitness, robots from Evolution-Only are suffering from local optimum. In these 10 robots, we observe a same morphology from both methods (cross-shape), however the robot from Evolution + Learning has a much higher fitness value, once again it shows the importance of matching body and brain. A video showing examples of robots from both types of experiments can be found in https://youtu.be/4RJBpdNIR30.

#### 4.2.2 Learning Delta

**FIGURE 14:**
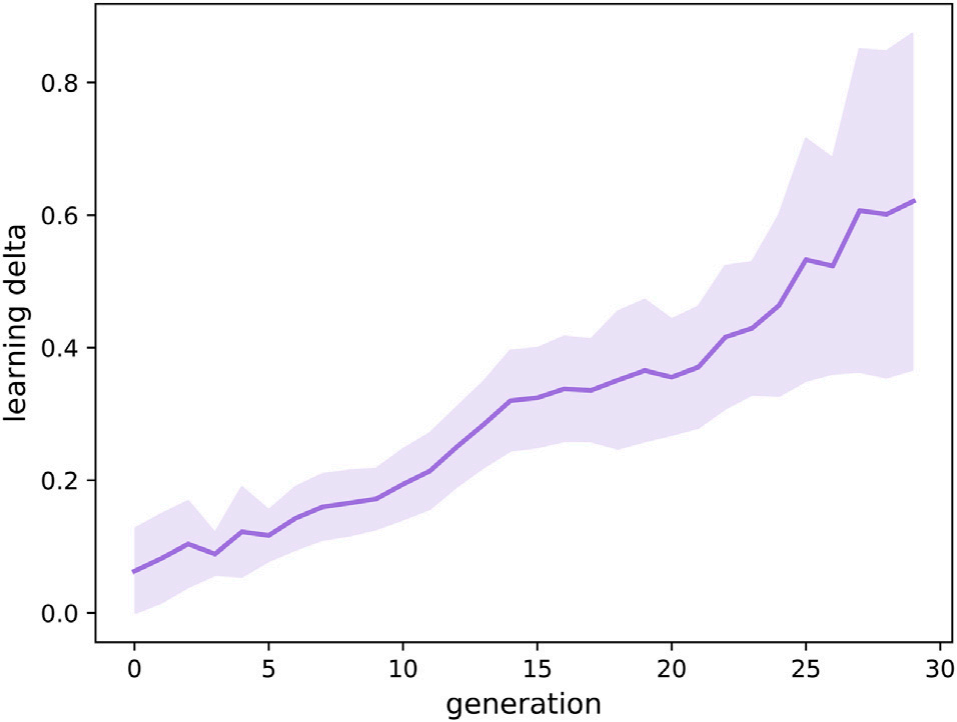
Progression of the learning Δ, the difference between the robots performance with its learned brain and the performance with its inherited brain, over the course of evolution averaged over 10 runs. The plot illustrates that the learning delta is growing across generations.

In this paper we also consider a special property of a robot morphology, its learning potential. Specifically, we measure the learning delta, the difference between the robots performance right after birth using its inherited brain and the performance after the learning process using the learned brain. Given a learning method and a learning budget (the number of learning trials) which are the same for all generations, the learning delta shows how well a given body facilitates the brain to achieve high task performance (fitness) through learning. In Figure 14, we see that the average learning delta grows across the generations. The growth is very steady. This effect has been discovered previously in (Miras et al., 2020), with a different task, a different learning method and a different representation, so the current results provide additional support that life-time learning leads the evolutionary search towards morphologies with increasing learning potential.

### 4.3 Behaviour

**FIGURE 15:**
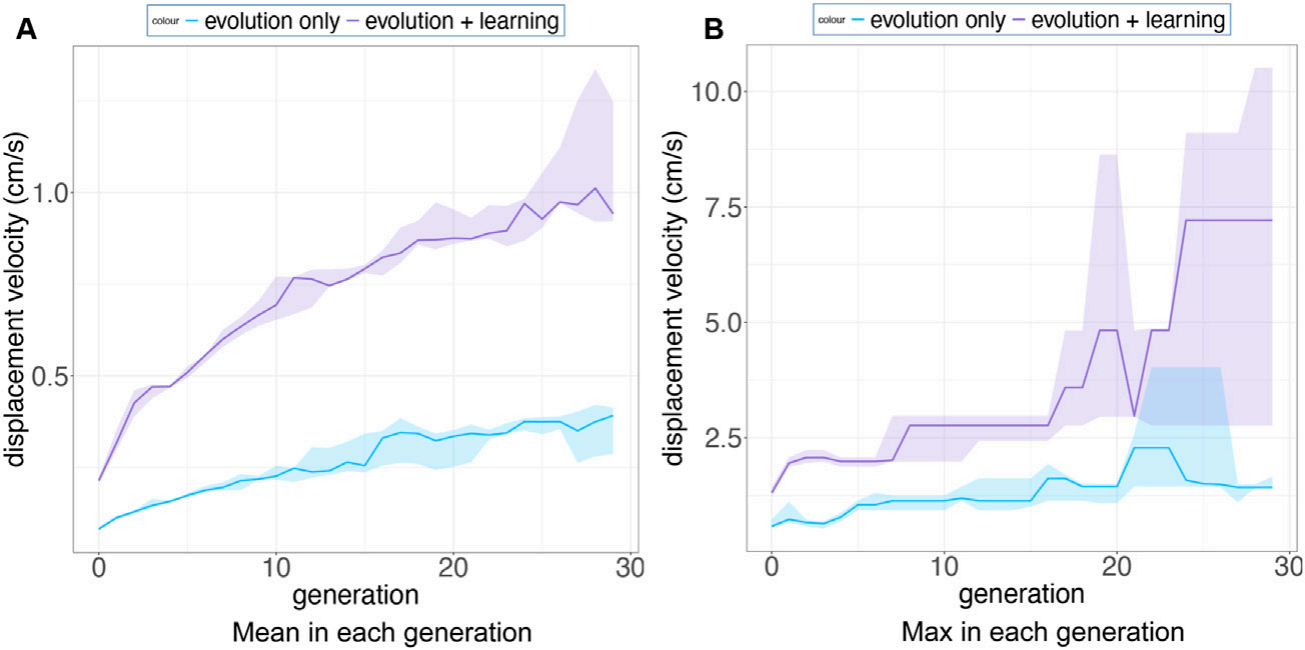
Subfigure **(A)** shows the mean displacement velocity over 30 generations (averaged over 10 runs) for Evolution-Only in blue and Evolution + Learning in purple. Subfigure **(B)** exhibits the maximum displacement velocity in each generation (averaged over 10 runs). The shaded areas show the standard deviation. Note the different scales on the vertical axes.

**FIGURE 16:**
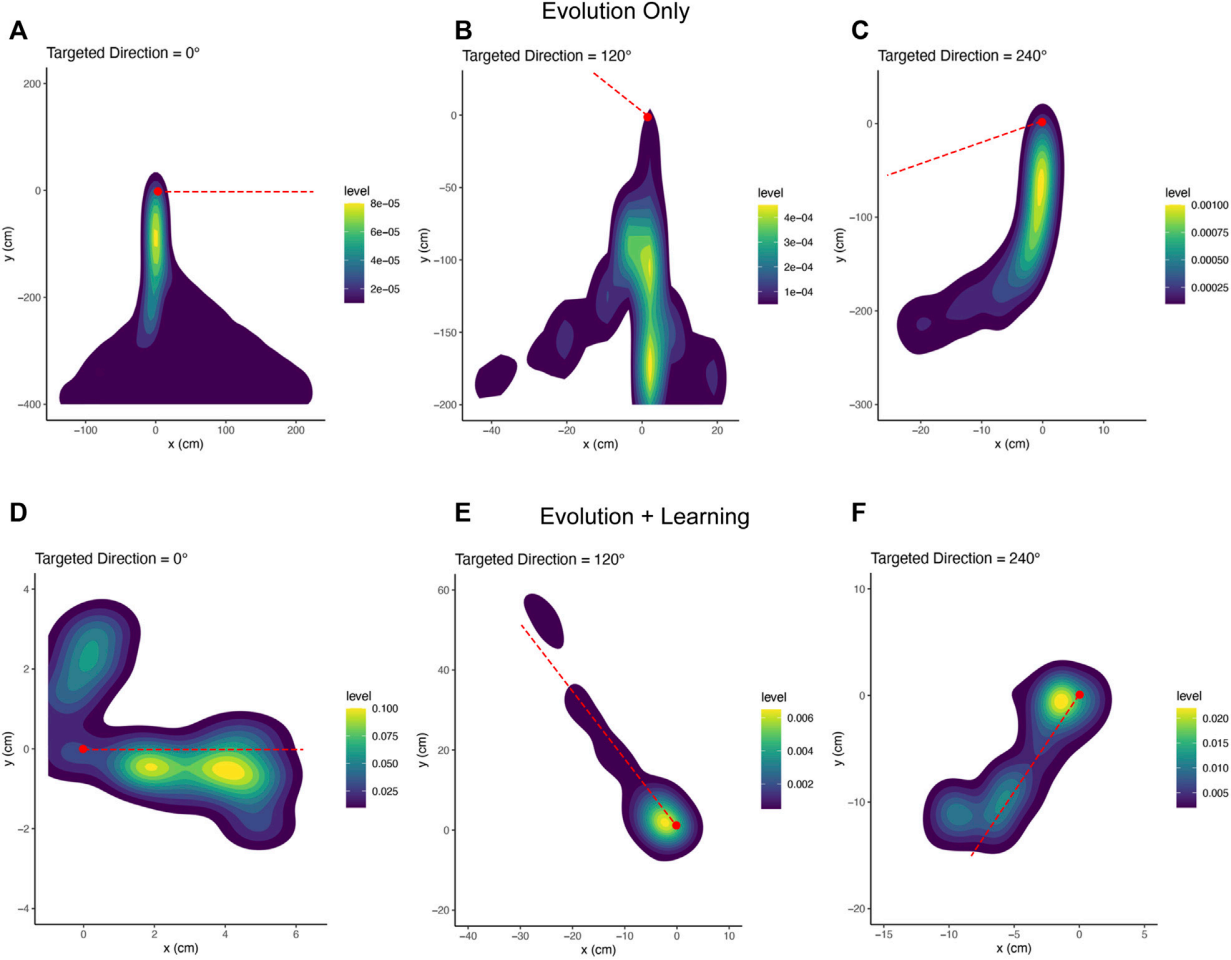
Trajectory density plots of the robots with maximum fitness in the last generation of all runs. The red dashed lines are the targeted directions. The red dots are the starting points.

Figure 15 shows that both the mean and maximum displacement velocities of these two methods are significantly different. Especially in Figure 15. b, the maximum displacement velocity in each generation from Evolution + Learning is much higher than Evolution-Only, it can be due to the shorter path length, or the higher speed. To verify the reason, we inspect the trajectories of the robots. In order to have a picture of several robots’ behavior rather than regarding individuals, we use a density plot to visualize the trajectories of the highest fitness robots in the last generation of all runs (Figure 16). In Figures 16A–C, we can observe that the best robots from Evolution-Only in a given time travelled a long distance, indicating a high speed, however they are not efficiently moving towards the target directions. In Figures 16D–F, we can notice that the best robots from Evolution + Learning travelled a shorter distance compared to Evolution-Only but very efficiently towards the targeted directions.

## 5 Conclusion and Future Work

The data on the efficacy measurements (Figures 7, 8) shows that Evolution + Learning can achieve much higher levels of fitness with the same number of fitness evaluations as Evolution-Only, the differences are very large. This renders the issue of efficiency quite irrelevant as it does not make much sense to compare the number of evaluations needed to achieve the same level of fitness.

These outcomes provide an answer to our first research question clearly showing that spending time on infant learning can lead to massive savings in terms of evolutionary trials; Evolution + Learning achieved speed levels in eight generations that required 21 generations for Evolution-Only. One could argue that this is not a fair comparison because the number of evaluations is much higher for Evolution + Learning. However, recall our considerations in the Introduction and in Section 4.1 about the great difference between the effort to make and test a new robot and the effort to make and test a new controller.

All in all, we can formulate a recommendation for users and experimenters: it is advisable to divide the number of allowable fitness evaluations between evolutionary trials and learning trials instead of spending them all on Evolution-Only. The balance, i.e., the adequate number of learning trials per newborn robot, will differ by the specific numbers of the given application, but our results demonstrate that the Triangle of Life is not only a concept of theoretical interest, but a methodology with practical benefits.

Regarding the second research question, the data on the morphological traits proved that the learning approach leads to differently evolved morphologies. This is highly interesting if we realize that the difference between Evolution-Only and Evolution + Learning is only affecting the controllers. In other words, our results show that modifying the way brains are treated results in pronounced changes to the bodies. Thus, we clearly demonstrate how the brains can shape the bodies through affecting task performance that in turn changes the fitness values that define selection probabilities during evolution. This is an interesting inversion of the classic statement that the bodies shape the brains (Pfeifer and Bongard, 2007).

Our third research question concerns the evolution of the learning delta. The crucial plot to this question is Figure 14 that shows how the delta is increasing over the course of evolution. This is a powerful demonstration of a how consecutive generations are becoming better and better learners which in turn makes them better and better at the given task. Putting it differently, evolution is producing robots with an increasing plasticity. Of course, we are not claiming that the learning delta values can grow without limits, but in the curve of the thirty generations we could afford with the current computing hardware there is no sign of a plateau.

In our view, this phenomenon calls for more research and elaboration. One possible way to interpret our learning delta is considering it as an indicator of morphological intelligence, because it is a property of the morphologies that is often associated with intelligence: the ability to learn and become good at the task at hand. Such a definition and that of (Gupta et al., 2021) are similar in spirit, although (Gupta et al., 2021) define morphological intelligence by the ability to learn novel tasks. Obviously, intelligence is a general and fundamental notion with several possible definitions and the same holds for morphological intelligence. We hope that our paper will contribute to a broader discussion regarding various facets of intelligence rooted in the morphologies, the controllers and the ways they are integrated.

For future work, we will study more tasks related to locomotion and object manipulation not only in isolation, but also in combination, such that multiple task-abilities determine the fitness together. Furthermore, we will investigate Lamarckian evolution, where the learned brain features are coded back to the genotypes, thus becoming inheritable. To this end we have to carefully consider a suitable genetic representation which allows us to partly invert the genotype-phenotype mapping of the brains.

## Data Availability

The original contributions presented in the study are included in the article/Supplementary Materials, further inquiries can be directed to the corresponding author.

## Footnotes

1 For the sake of simplicity, here we call all tests a fitness evaluation.
